# miRNA *let-7-5p* present in the extracellular vesicles of *Trichinella spiralis* newborn larvae inhibits the function of M1-type RAW264.7 macrophages by targeting *C/EBPδ*

**DOI:** 10.1186/s13071-025-06802-2

**Published:** 2025-06-01

**Authors:** Yi Liu, Yu Chun Cai, Jia Xu Chen, Shao Hong Chen, Ying Fang Yu

**Affiliations:** 1 National Institute of Parasitic Diseases, Chinese Center for Disease Control and Prevention (Chinese Center for Tropical Diseases Research); National Key Laboratory of Intelligent Tracking and Forecasting for Infectious Diseases; National Health Commission Key Laboratory On Parasite and Vector Biology, WHO Collaborating Centre for Tropical Diseases, National Center for International Research on Tropical Diseases, Ministry of Science and Technology, Shanghai, 200025 China; 2 Shanghai University of Medicine & Health Science, The College of Medical Technology, Shanghai, 201318 China

**Keywords:** *Trichinella spiralis*, Newborn larvae, Extracellular vesicles, Macrophages, *let-7-5p*, *C/EBPδ*

## Abstract

**Background:**

*Trichinella spiralis*, in its newborn larva (NBL) stage, invades the host bloodstream and disseminates throughout the body. Concurrently, M1 macrophages undergo transformation into M2 macrophages. In our previous studies, we demonstrated that extracellular vesicles secreted by NBL (NBL-EVs) significantly express the microRNA (miRNA) *cel-let-7-5p*. In this study, we investigated the immunomodulatory effects and mechanisms of action of EVs derived from *T. spiralis* NBL and the influence of their key miRNA, *cel-let-7-5p*, on M1 macrophages.

**Methods:**

This study investigates the impact of *T. spiralis* NBL-EVs and *cel-let-7-5p* on RAW264.7 macrophages through in vitro co-culture, followed by a dual luciferase assay to confirm *C/EBPδ* as the target of *cel-let-7-5p*. M1-polarized RAW264.7 cells were subsequently transfected with various agents, including NBL-EVs, *cel-let-7-5p* mimic, *C/EBPδ* small interfering RNA (siRNA), and so forth. The cell functions, surface molecule expression, transcription, and cytokine release were analyzed using flow cytometry, reverse transcription polymerase chain reaction (RT-PCR), western blot, and enzyme-linked immunosorbent assay (ELISA) to elucidate the regulatory mechanisms of NBL-EVs and *cel-let-7-5p* on macrophage polarization.

**Results:**

Results show that *cel-let-7-5p* transported by *T. spiralis* NBL-EVs inhibited the functional activity of M1 RAW264.7 macrophages by targeting* C/EBPδ*. This inhibition was validated by reduced CD86 and increased CD206 expression, along with decreased nitric oxide (NO) synthesis and downregulation of the M1 marker genes interleukin-12 (*IL-12*) and inducible nitric oxide synthase (*iNOS*). In contrast, the messenger RNA (mRNA) levels of *IL-10* and arginase-1 (*Arg1*), which are M2 characteristic genes, were significantly enhanced. However, the release of M1 pro-inflammatory cytokines, such as IL-6, tumor necrosis factor-alpha (TNF-α), and IL-1β, was decreased proportionally. Notably, introducing a *cel-let-7-5p* inhibitor effectively reversed the suppressive effect of NBL-EVs on M1 macrophage function and partially mitigated their transition to the M2 phenotype, notably impacting *Arg1* gene expression. However, no significant changes were observed in CD206 protein expression or *IL-10* mRNA levels.

**Conclusions:**

The findings of this study reveal that *cel-let-7-5p* in *T. spiralis* NBL-EVs can inhibit the function of M1-type RAW264.7 macrophages by targeting *C/EBPδ*.

**Graphical Abstract:**

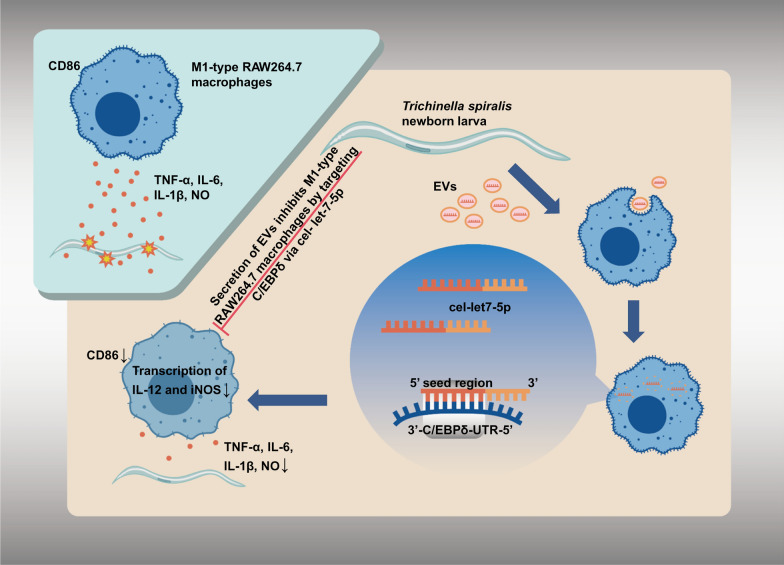

## Background

Trichinellosis is a parasitic disease that affects humans and vertebrates. Infection by *Trichinella spiralis* is primarily acquired by ingesting raw or uncooked meat products that contain its larvae [1]. The life cycle of *T. spiralis* includes the adult and larval stages, with the larval stage further subdivided into newborn larvae (NBL) and muscle larvae (ML). NBL is a crucial phase for the parasite dissemination in the host body; it is the only stage capable of migrating through the bloodstream during the entire life cycle; therefore, it is crucial for triggering a protective immune response in the host [2]. In-depth studies on the dynamics between the NBL and host immune defenses may contribute to a more comprehensive understanding of the mechanisms underlying the parasite–host relationship, possibly leading to the development of more potent measures for trichinellosis prevention and treatment than conventional therapies.

Macrophages are versatile cells that are capable of phagocytosing pathogens, infected cells, debris, and dead cells. They also function as antigen-presenting cells and produce various cytokines. They can polarize and form distinct subpopulations in different tissues depending on environmental changes [3]. These cells are bifurcated into M1 and M2 states, which are characterized by the differential expression of surface markers, release of specific cytokines, and unique biological activities. Both phenotypes secrete various cytokines and inflammatory mediators that regulate immune responses and inflammatory reactions. They are characterized by plasticity and multifunctionality, adjusting their functions in response to external environmental signals [4]. Additionally, they are activated by T helper cell 1 (Th1)-derived cytokines, such as interferon-gamma (IFN-γ) and tissue necrosis factor (TNF)-α, or by bacterial lipopolysaccharide (LPS). M1 macrophages produce high levels of inflammatory cytokines, including tumor necrosis factor alpha (TNF-α), interleukin (IL)-1α, IL-1β, IL-6, IL-12, IL-23, and cyclooxygenase-2 (COX-2), whereas they produce low levels of IL-10. They trigger the nicotinamide adenine dinucleotide phosphate (NADPH) oxidase pathway and generate reactive oxygen species (ROS) to eliminate pathogens and combat infections. However, ROS generation causes tissue damage, which potentially disrupts tissue regeneration and healing mechanisms, eventually reducing chronic inflammatory responses [5]. In contrast, M2 macrophages possess anti-inflammatory properties and are further classified into subtypes M2a, M2b, M2c, and M2d. In this classification, the IL-4 and IL-13-induced M2a macrophages release IL-10, transforming growth factor beta (TGF-β), chemokine (C–C motif) ligand (CCL) 17, CCL18, CCL22, and CCL24. These products suppress inflammatory responses and coordinate tissue remodeling. M2 macrophages are crucial for the development of protective responses during parasitic worm infections, owing to their crucial roles in infection-related and post-infection tissue repair and remodeling [6].

Sun *et al*. [7] studied the dynamic changes in macrophage subsets within mouse mesenteric lymph nodes, spleen, intestine, and muscle on days 1, 5, 15, and 30 post-infection with *T. spiralis*. Their findings revealed that the transition of macrophages from the M1 to M2 phenotype occurred at the same time as the migration of NBL within the host. This suggests that during *Trichinella* infection, NBL produced by adult worms in the intestine invade the lymphatic system through small intestinal epithelial cells and travel in the bloodstream to the muscle tissue, causing severe inflammation and tissue damage in the host. During infection, host macrophages differentiate into the M1 phenotype and secrete large amounts of pro-inflammatory cytokines to mediate the Th1 immune response for the eradication of *Trichinella*. For example, macrophages release nitric oxide (NO) and mediate antibody-dependent cytotoxicity (ADCC) to directly eliminate *Trichinella* NBL [8]. Furthermore, *Trichinella* cathepsin L induces macrophage polarization toward an M1 phenotype through the nuclear factor kappa B (*NF-κB*) pathway, thereby increasing ADCC-mediated NBL destruction [9]. To resist host clearance, *Trichinella* highly expresses dipeptidyl peptidase 1 during the intestinal infective larval (IIL) and adult stages. This promotes macrophage polarization toward an M2 phenotype through the signal transducer and activator of transcription 6 (*STAT6*)/peroxisome proliferator-activated receptor gamma (*PPARγ*) pathway and inhibits the cytotoxicity of M1 macrophages [10]. *Trichinella* NBL and ML suppress M1 macrophages, thereby promoting the transition toward M2 polarization and inhibiting anti-inflammatory and Th1 immune responses, as the NBL gradually develop into ML and form cysts. This promotes the healing of damaged tissues and evades clearance by the host immune system.

Notably, extracellular vesicles (EVs), which are lipid-bilayer-enclosed particles emitted by cells, lack the capacity for self-replication [11]. Proteins, lipids, and nucleic acids constitute the biomolecules encapsulated within EVs. Helminth EVs modulate immune responses by regulating host immune cell function and cytokine secretion [12], serving as crucial tools for intercellular communication during immunoregulation. EVs transport genomic DNA, messenger RNA (mRNA), and non-coding RNAs, particularly microRNAs (miRNAs), thereby providing a novel mechanism for gene exchange. miRNAs are small non-coding RNAs with significant genetic regulatory capacity. They regulate signaling pathways by interacting with target genes. Additionally, they recognize homologous sequences and influence gene expression at the transcriptional, translational, and epigenetic levels [13–15]. Recent studies have demonstrated that EVs mediate the immunoregulatory effects of *T*. *spiralis* on macrophages, especially EVs secreted by ML (ML-EVs), which inhibit M1 macrophages [16]. However, the mechanism through which EVs secreted by NBL (NBL-EVs) regulate macrophages remains unclear. In our previous study, we reported that NBL-EVs express* cel-let-7-5p* at significantly higher levels than NBL [17]. The miRNA *let-7* family is highly conserved across multiple species and participates in biological development and disease progression through targeted mRNA regulation [18]. Recently, *let-7-5p* in *T. spiralis* ML-EVs was reported to promote bone marrow macrophage polarization toward the M2b phenotype and inhibit fibroblast activation [19]. *Let-7-5p* originating from *Taenia solium* and *T. crassiceps,* upon activation, substantially suppresses macrophage secretion of pro-inflammatory cytokines, including IL-16, IL-12, and TNF [20]. Moreover, a study has clarified that *let-7-5p* derived from *Taenia pisiformis* cysticercus EVs induces a shift toward the M2 macrophage phenotype by regulating CCAAT/enhancer binding protein delta (*C/EBPδ*), a key transcription factor in macrophage polarization [21]. We hypothesized that *T. spiralis* NBL-EVs may exhibit similar regulatory effects on macrophages, given the high conservation of *let-7-5p* across different species.

In this study, we investigated the immunomodulatory effects and mechanisms of action of EVs derived from *T. spiralis* NBL and the influence of their key miRNA, *cel-let-7-5p*, on M1 macrophages. We performed in vitro co-culture studies to evaluate the effects of NBL-EVs and *cel-let-7-5p* on the murine macrophage RAW264.7 cell line. Additionally, a dual luciferase reporter assay was used to confirm that *C/EBPδ* is the target of *cel-let-7-5p*. Finally, cellular function assays were performed to examine the specific effects of *cel-let-7-5p* on the functionality, molecular expression, and cytokine secretion of M1 RAW264.7 macrophages. These findings enhance our understanding of larval invasion mechanisms during *T. spiralis* infection and provide a scientific basis for developing new therapeutic strategies, including vaccines and drugs.

## Methods

### NBL-EVs and cell line

NBL-EVs were isolated from the NBL culture supernatant of a Chinese Henan *T. spiralis* isolate (International Standard Number: ISS534) using differential ultracentrifugation. In accordance with the minimal information for studies of extracellular vesicles 2023 (MISEV2023) criteria, we confirmed the reliability and purity of NBL-EVs through transmission electron microscopy, nanoparticle tracking analysis, and mass spectrometry [17]. The murine macrophage cell line RAW264.7 was a kind gift from the Cell Bank of the Chinese Academy of Sciences. However, the human embryonic kidney cell line 293T (HEK293T) sourced from Shanghai OBiO Biotechnology Co., Ltd., was used in this study.

### Synthesis of small interfering RNA (siRNA) mimics

The siRNA and negative control (NC) mimics and inhibitor mimics were synthesized by Shanghai OBiO Biotechnology Co., Ltd. (Table 1).

**Table 1:** The siRNA used in this study

Name	miRNA mimics	Sequence	
Mimics	*cel-let-7-5p* mimics	Sense strand (5′–3′):	UGAGGUAGUAGGUUGUAUAGUU
		Antisense strand (5′–3′):	CUAUACAACCUACUACCUCAUU
Mimics-NC	*cel-let-7-5p* mimics-NC	Sense strand (5′–3′)	UUCUCCGAACGUGUCACGUdTdT
		Antisense strand (5′–3′):	ACGUGACACGUUCGGAGAAdTdT
Inhibitor	*cel-let-7-5p* inhibitor	Strand (5′–3′):	AACUAUACAACCUACUACCUCA
Inhibitor-NC	*cel-let-7-5p* inhibitor-NC	Sense strand (5′–3′)	UUCUCCGAACGUGUCACGUdTdT
		Antisense strand (5′–3′):	ACGUGACACGUUCGGAGAAdTdT
siRNA	*C/EBPδ* siRNA	Sense strand (5′–3′):	CCGACCUCUUCAACAGCAACC
		Antisense strand (5′–3′):	UUGCUGUUGAAGAGGUCGGCG
si-NC	*C/EBPδ* siRNA NC	Sense strand (5′–3′)	UUCUCCGAACGUGUCACGUTT
		Antisense strand (5′–3′):	ACGUGACACGUUCGGAGAATT

NC, negative control; *C/EBPδ*, CCAAT/enhancer binding protein delta; si-NC; small interfering RNA

### Uptake of NBL-EVs by RAW264.7 cells

To label NBL-EVs with PKH67 dye, we diluted 4 μl of the dye in 500 μl of diluent C from the PKH67 staining kit (Sigma-Aldrich, St. Louis, MO, USA), and mixed thoroughly before setting it aside. We diluted the NBL-EVs to a final volume of 500 μl using diluent C, mixed thoroughly, and subsequently combined it with the diluted dye. For staining, the cells were incubated at ambient temperature for 5 min. The reaction was quenched by adding 1% bovine serum albumin (BSA), followed by centrifugation at 120,000×*g* for 60 min. The supernatant was removed, and the pellet was resuspended in sterile phosphate-buffered saline (PBS).

We began the preparation of RAW264.7 cells by washing, trypsinization, and centrifugation, and discarding the supernatant. The cells were resuspended in a culture medium comprising 89% Dulbecco’s modified Eagle’s medium (DMEM) high-glucose medium (HyClone, South Logan, UT, USA), 10% fetal bovine serum (FBS) (Gibco, Grand Island, NY, USA), and 1% penicillin–streptomycin (Gibco, Grand Island, NY, USA). The cells were seeded in a 24-well plate and cultured overnight. On the following day, we replaced the supernatant with the fresh complete medium of 10 μg/ml PKH67-labeled NBL-EVs per well, followed by incubation for an additional 24 h. After incubation, the wells were washed with PBS, fixed with 4% paraformaldehyde, washed, permeabilized with 0.1% Triton X-100 (Beyotime), and washed with PBS. The cells were mounted on coverslips using a DAPI-containing mounting medium (Beyotime, Shanghai, China) and examined under an SP9 laser confocal microscope (Leica, Wetzlar, Germany).

### Cell counting kit-8 (CCK-8) assay

The experiment included four groups: a control group without treatment (blank cell group), the EV group, the miRNA *cel-let-7-5p* mimic group, and the non-specific mimic control (mimics-NC) group. Each group comprised six replicates. Mouse leukemic monocyte-macrophage cells (RAW264.7) were thawed and cultured in a complete medium. Subsequently, cell growth was monitored for 24 h after seeding. The cultures were passaged when the monolayer reached 80% confluence. Next, the cells were rinsed with PBS, digested with trypsin-ethylenediaminetetraacetic acid to produce a single-cell solution, and placed in six-well plates. Attaining approximately 80% confluence the following day, the cells were subjected to transfection with Lipo 8000 (Beyotime, Shanghai, China) at room temperature for 6 h. After diluting NBL-EVs to a concentration of 50 μg/ml using DMEM, the medium in the six-well plates was exchanged for this diluted preparation. The EV group received this concentration of NBL-EVs, whereas the other groups received only the Lipo 8000 reagent mixture and were cultured for 48 h. Subsequently, the cells were transferred to a 96-well plate and cultured overnight. Cell viability was assessed using the CCK-8 assay kit (Beyotime, Shanghai, China). After the introduction of CCK-8, the cells were incubated in the dark for 4 h, and the optical density (OD) at 450 nm was determined using a SpectraMax i3 plate reader (Molecular Devices, San Jose, CA, USA) to calculate the cell survival rate.

### Experimental grouping, cell transfection, and culture

The experiment comprised nine groups: (1) M0 group, (2) M1 group, (3) M1+EVs group, (4) M1+mimics group, (5) M1+mimics-NC group, (6) M1+EVs+inhibitor group, (7) M1+EVs+inhibitor-NC group, (8) M1+siRNA, and (9) M1+si-NC. Each group comprised three replicates. The differentiation of M0 RAW264.7 macrophages was primarily triggered by co-stimulation with a high-dose LPS solution (1 mg/ml; Sigma-Aldrich, USA) in combination with 10 ng/ml IFN-γ (T&L Biotechnology, Beijing, China). This potent cytokine cocktail was deliberately employed to establish a sustained pro-inflammatory M1 phenotype, thus generating an optimized in vitro system that amplifies detection sensitivity for evaluating NBL-EV-mediated anti-inflammatory effects through enhanced polarization contrast. Furthermore, M0 and M1 cells were cultured in six-well plates until they reached approximately 80% confluence. The following day, the transfection mixtures were prepared with 100 pmol of each agent (mimics, mimics-NC, inhibitor, inhibitor-NC, siRNA, and si-NC), combined with 375 µl of DMEM without antibiotics and serum, and 12 µl Lipo 8000. NBL-EVs were diluted to reach a concentration of 50 µg/ml. Fresh complete medium was used to replace the cultured cells in the six-well plate. Subsequently, the M1 mouse macrophage RAW264.7 cells were treated with various reagents to establish experimental groups, including *T. spiralis* NBL-EVs, *cel-let-7-5p* mimics, *cel-let-7-5p* mimics-NC, EVs + *cel-let-7-5p* inhibitor, EVs + *cel-let-7-5p* inhibitor-NC, *C/EBPδ* siRNA, and *C/EBPδ* siRNA NC. For the M1+EVs, M1+EVs+inhibitor, and M1+EVs+inhibitor-NC groups, 50 µl of EVs was added to each well followed by 125 µl of the respective transfection mixture. After culturing for an additional 24 h, we collected the supernatant and cell samples from each group for subsequent analyses.

### Flow cytometry

The samples from each group were digested with 0.25% trypsin (Gibco) at room temperature for 10 min. After centrifugation at 150×*g* for 15 min, pellets were collected. The cells were then washed three times with PBS and resuspended at a density of 1 × 10^5^ cells/ml. Aliquots of 100 μl from each suspension were incubated with anti-mouse CD206-phycoerythrin (CD206-PE) and CD86 fluorescein isothiocyanate (CD86-FITC) antibodies (eBioscience, San Diego, CA, USA) in the dark for 15 min at 24 °C. This was followed by washing and resuspension in 100 μl PBS. CD86 and CD206 expression levels were determined using a FACSVerse flow cytometer (BD Biosciences, San Jose, CA, USA), and FlowJo software (BD Biosciences) for data analysis.

### Nitric oxide content detection

Supernatants from each culture group were collected for the NO release analysis, which was performed using a NO content detection kit (Beyotime), following the manufacturer’s guidelines. Sample nitrite levels were determined by measuring the absorbance at 540 nm using a SpectraMax i3 microplate reader and referenced against a standard curve for calculation.

### Enzyme-linked immunosorbent assay (ELISA)

Following the manufacturer’s instructions, mouse TNF-α, IL-6, and IL-1β kits (mlbio, Shanghai, China) were used to assess TNF-α, IL-1β, and IL-6 in the supernatants of cultured cells. The optical density (OD) at 450 nm was quantified using a SpectraMax i3 microplate reader, and cytokine concentrations were calculated based on the standard curves for each cytokine.

### Reverse transcription polymerase chain reaction (RT-PCR)

We used RT-PCR to evaluate the transcriptional activity of phenotypic markers *C/EBPδ*, *IL-12*, inducible nitric oxide synthase (*iNOS*), *let-7-5p*, *IL-10*, and arginase-1 (*Arg1*) across different cell groups. Total RNA was extracted using TRIzol reagent (Invitrogen, Carlsbad, CA, USA), and reverse transcription was performed using a First Strand cDNA [complementary DNA] Synthesis Kit (Aidlab Biotechnologies, Beijing, China). PCR amplifications were performed on a 7500 Real-Time PCR machine (Applied Biosystems, Foster City, CA, USA), with *GAPDH* as the reference gene for mRNA and circular RNA (circRNA), and *U6* for *let-7-5p*. Primers specified in Table 2, synthesized by Shanghai OBiO Biotechnology Co., Ltd., were used. The PCR program commenced with an initial denaturation at 95 °C for 10 min, followed by 40 cycles at 95 °C for 20 s, 55 °C for 20 s, and 72 °C for 20 s, and ended with a melting curve analysis at 95 °C for 15 s, 60 °C for 60 s, and 95 °C for 15 s. The 2^−ΔΔCT^ method was used to ascertain the quantitative changes in gene expression.

**Table 2:** Primers used for RT-PCR analysis

Primers	Sequence (5′–3′)
*cel-let-7-5p*-F	ACACTCCAGCTGGGTGAGGTAGTAGGTTGT
*cel-let-7-5p*-R	CTCAACTGGTGTCGTGGAGTCGGCAATTCAGTTGAGAACTATAC
*C/EBPδ*(mouse)-RT-F	CATGTACGACGACGAGAG
*C/EBPδ*(mouse)-RT-R	TGGTTGCTGTTGAAGAGG
Unified reverse primer	TGGTGTCGTGGAGTCG
*U6*-F	CTCGCTTCGGCAGCACA
*U6*-R	AACGCTTCACGAATTTGCGT
(mouse)-RT-F	CAGGTGTCTTAGCCAGTC
*Il-1*2(mouse)-RT-R	CTCTCGTTCTTGTGTAGTTC
*Il-10*(mouse)-RT-F	GGTTGCCAAGCCTTATCG
*Il-10*(mouse)-RT-R	TCTTCACCTGCTCCACTG
*INOS*(mouse)-RT-F	TACTGCTGGTGGTGACAA
*INOS*(mouse)-RT-R	CTGAAGGTGTGGTTGAGTT
*Arg1*(mouse)-RT-F	AAGGTCTCTACATCACAGAAG
*Arg1*(mouse)-RT-R	CGAAGCAAGCCAAGGTTA
*GAPDH*(mouse)-RT-F	GGTGAAGGTCGGTGTGAACG
*GAPDH*(mouse)-RT-R	CTCGCTCCTGGAAGATGGTG

### Dual luciferase reporter gene assay

Based on a previous study [21], the mouse macrophage transcription factor *C/EBPδ* was selected as the target gene for *cel-let-7-5p*. Using the mouse *C/EBPδ* gene (ID: 12609) retrieved from the National Center for Biotechnology Information (NCBI) database, Jiangsu Saisife Biotechnology Co., Ltd. was commissioned to construct recombinant plasmids containing wild-type and mutant mRNA *C/EBPδ*-3′ untranslated region (UTR) using the psiCHECK2.0 vector, which were employed to verify  *cel-let-7-5p* target genes via a dual luciferase reporter system. Recombinant plasmids containing *let-7-5p* mimics and non-specific mimic controls (mimic NC) were introduced into HEK-293 T cells, which were subsequently cultured for 24 h. Luciferase activity in the cells was measured using the Dual-Luciferase Reporter Assay System (Promega, Madison, WI, USA) following the manufacturer’s instructions, and readings were obtained using a SpectraMax i3 Microplate Reader.

### Western blot analysis

Western blotting was used to quantify the intracellular expression of C/EBPδ and GAPDH proteins. The cells were harvested, trypsinized, and centrifuged. Radioimmunoprecipitation assay (RIPA) lysis buffer (Beyotime, Shanghai, China) with phenylmethylsulfonyl fluoride (PMSF) added at a ratio of 1:100 was used to lyse cells. After centrifugation at 12,000×*g* for 10 min at 4 °C, the supernatant was extracted. Protein concentration was measured using a BCA kit (Thermo Scientific, Waltham, MA, USA). A 10% sodium dodecyl sulfate–polyacrylamide gel electrophoresis (SDS-PAGE) was used to separate 30 μg of protein lysates, which were subsequently transferred to polyvinylidene fluoride membranes (Millipore, Bedford, MA, USA). The membranes were washed three times with Tris-buffered saline with Tween-20 (TBST) (Beyotime, Shanghai, China) for 5 min each and blocked with western blocking solution for 2 h. They were then incubated with primary antibodies against C/EBPδ at 1:1000 (Abcam, Cambridge, MA, USA) and glyceraldehyde-3-phosphate dehydrogenase (GAPDH) at 1:5000 (Proteintech, Wuhan, China) overnight at 4 °C. After three 1-min washes with TBST, the membranes were incubated with horseradish peroxidase-conjugated secondary antibodies at 1:5000 (ZSGB-BIO, Beijing, China) for 1 h at 37 °C. Following additional TBST washes, an enhanced chemiluminescence reagent (Share-Bio, Shanghai, China) was used, and the signal was detected using ChemiScope 5300 Pro (Clinx, Shanghai, China). Band intensities were quantified, and C/EBPδ grayscale values were normalized to GAPDH for relative protein expression levels.

### Statistical analysis

Data analysis was performed using SPSS Statistics software (version 26.0, IBM, Armonk, NY, USA). Results from the experiments are expressed as the mean ± standard deviation (mean ± SD). GraphPad Prism 9 (GraphPad Software, La Jolla, CA, USA) was used for the graphical representation of data. An independent-samples *t*-test was utilized to analyze the data from the dual luciferase assay, whereas a paired *t*-test was used for comparisons among groups in other tests. Statistical significance was defined as *P* < 0.05.

## Results

### *Trichinella spiralis* NBL-EVs can inhibit the viability of RAW264.7 cells

**Fig. 1:**
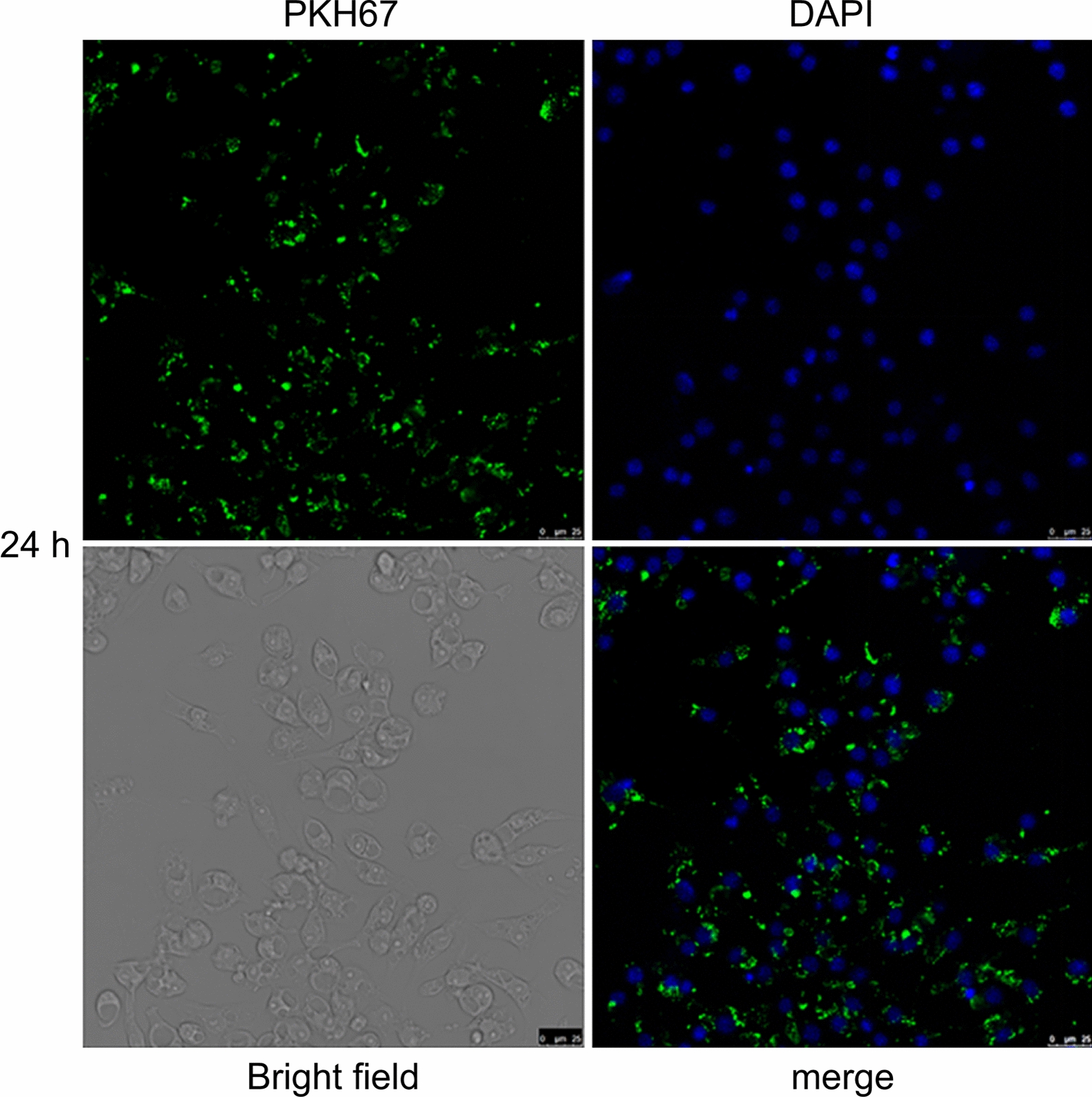
Results of co-incubation of RAW264.7 cells with PKH67-labeled *T. spiralis* NBL-EVs (×400 magnification). After labeling with PKH67, *T. spiralis* NBL-EVs showed green fluorescence. The nuclei of RAW264.7 cells stained with DAPI showed blue fluorescence. Light microscopy revealed the diverse morphological characteristics of RAW264.7, which appeared round, spindle-shaped, fusiform, or irregular with three or more protrusions. A merged image revealed that RAW264.7 cells effectively internalized PKH67-labeled *T. spiralis* NBL-EVs following a 24-h co-incubation period

**Fig. 2:**
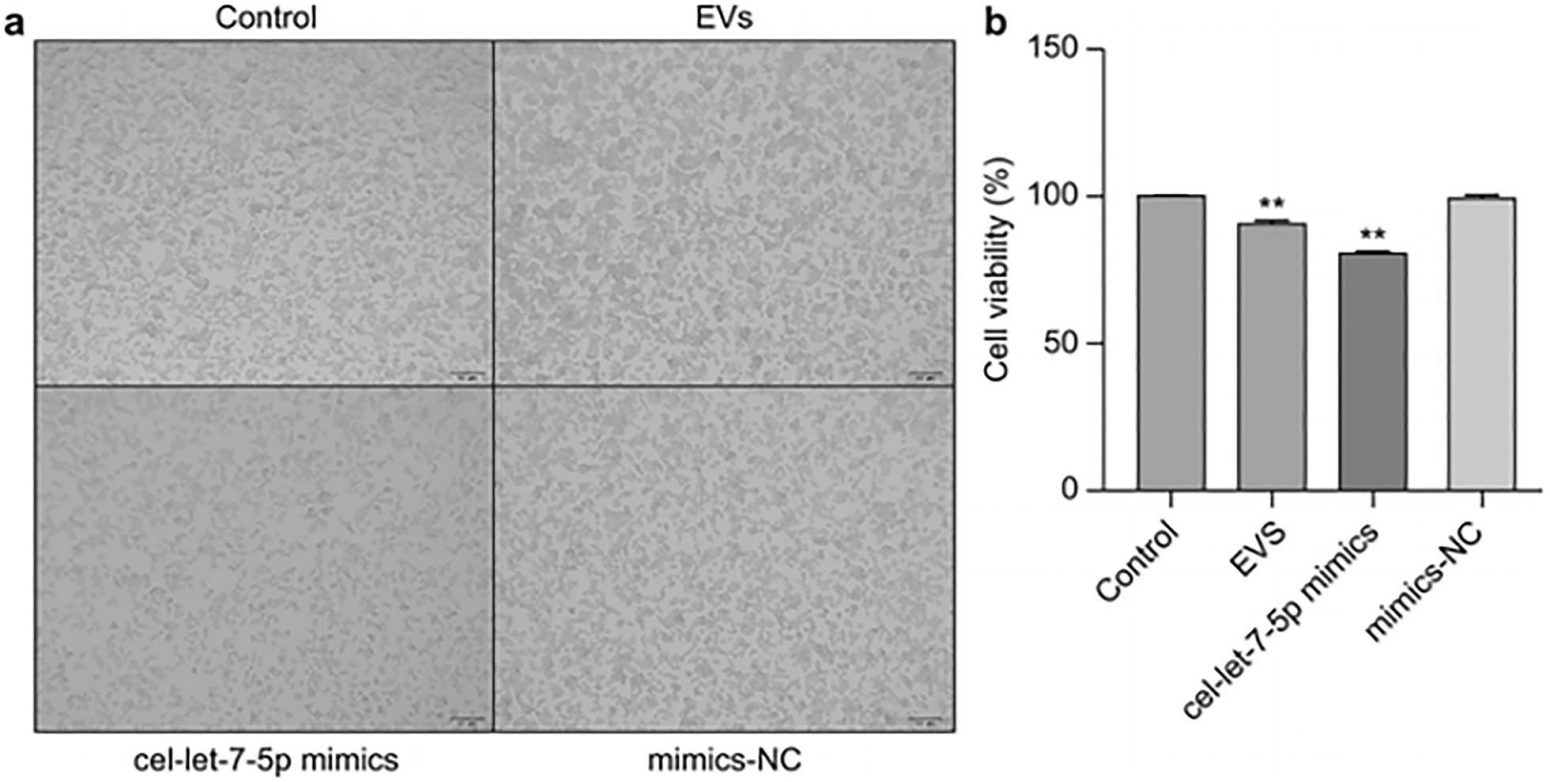
Impact of *T. spiralis* NBL-EVs and *cel-let-7-5p* on RAW264.7 macrophage viability assessed using CCK8. The morphology under a light microscope of the control, EVs, *cel-let-7-5p* mimic, and mimics-NC groups showed that the density of RAW264.7 cells in the EVs and those in *cel-let-7-5p* mimic groups decreased (×100 magnification). The cell viability of the EVs and *cel-let-7-5p* mimic groups also decreased significantly (*P* < 0.01). The data were analyzed from six independent experiments, and are expressed as the mean ± SD. ** *P* < 0.01, compared with the control group. EVs, extracellular vesicles; NC, negative control

The results showed that mouse macrophage RAW264.7 cells could internalize *T. spiralis* NBL-EVs (Fig. 1). The cell density in the EV-treated group decreased owing to the reduced proliferation after a 72-h co-culture with NBL-EVs (*P* < 0.01; Fig. 2). Cells transfected with the primary miRNA component of NBL-EVs, *cel-let-7-5p*, showed a significant decrease in viability (*P* < 0.01), whereas cell viability in the mimic-NC group remained unchanged (*P* > 0.05; Fig. 2). These results revealed that the suppressive effect of NBL-EVs on RAW264.7 cell viability could be attributed to the predominant miRNA component, *cel-let-7-5p*.

### Detection of macrophage surface marker expression by flow cytometry

**Fig. 3:**
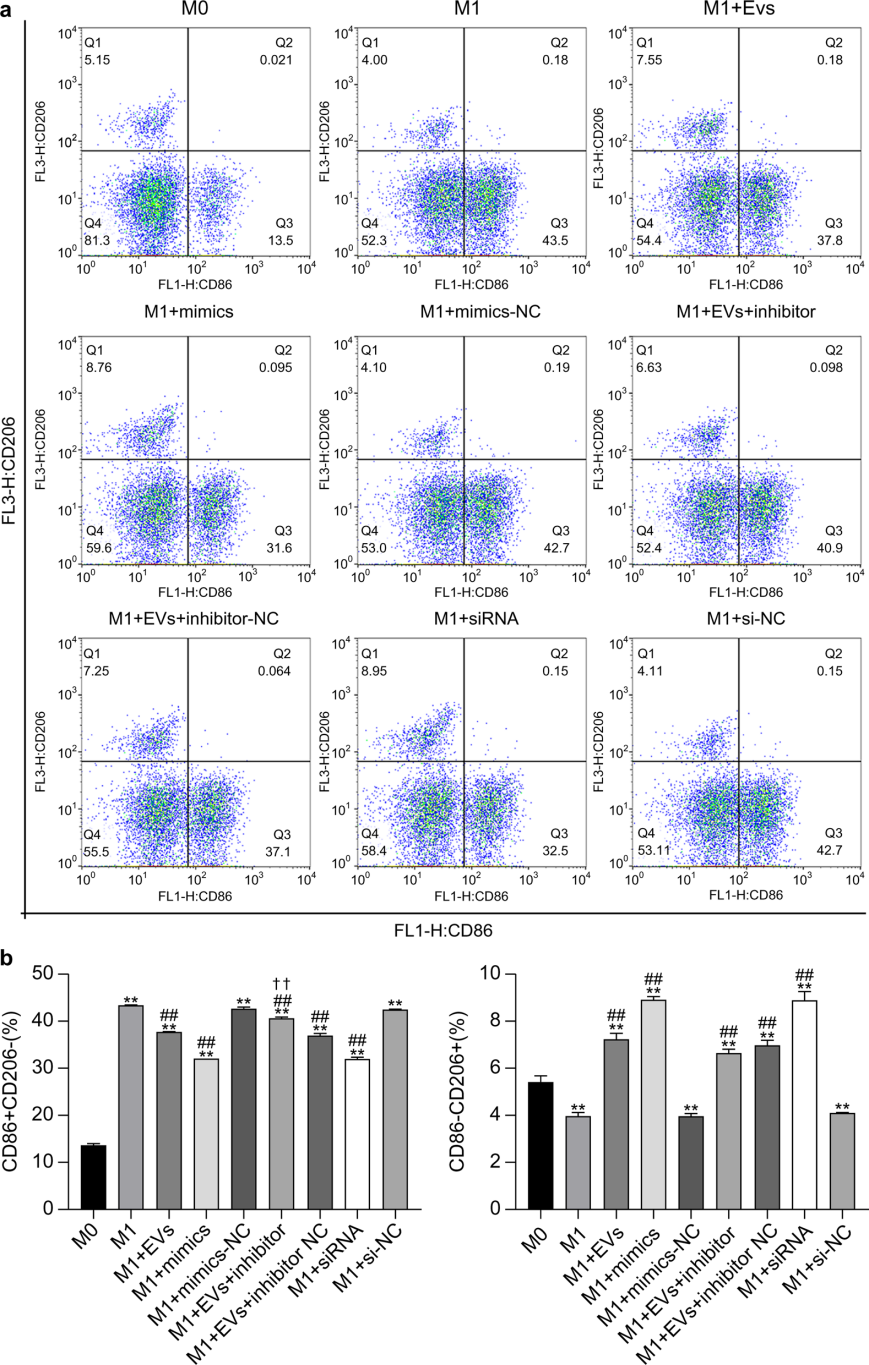
CD86/CD206 expression on RAW264.7 cells assessed via flow cytometry. **a** Flow cytometry scatter plot of CD86/CD206 expression on RAW264.7 cells. **b** Statistical histograms of CD86/CD206 expression on RAW264.7 groups. The data were analyzed from three independent experiments, and are expressed as the mean ± SD. **P < 0 .01, compared with the M0 group. ##*P* < 0.01, compared with the M1 group. ✝✝*P* < 0.01, compared with the M1+EVs group. EVs, extracellular vesicles; NC, negative control; siRNA, small interfering RNA; FL3-H, fluorescence detection channel 3-height; CD86, cluster of differentiation 86; CD206, cluster of differentiation 206

Analysis of CD86 and CD206 surface molecule expression on RAW264.7 cells in each group was performed using flow cytometry (Fig. 3a). The findings revealed that M1 cells had a significantly higher proportion of CD86^+^CD206^−^ cells (*P* < 0.01) and a significantly lower proportion of CD86^−^CD206^+^ cells (*P* < 0.01) than the M0 control group. Treatment with EVs, *cel-let-7-5p* mimics, EVs+inhibitor, EVs+inhibitor-NC, and small interfering RNA (siRNA) led to a significant reduction in CD86^+^CD206^−^ cells (*P* < 0.01) and a significant increase in CD86^−^CD206^+^ cells (*P* < 0.01) when compared with the M1 group. Moreover, the M1+EVS+inhibitor group had a significantly higher proportion of CD86^+^CD206^−^ cells than the M1+EVs group (*P* < 0.01); however, the reduction of CD86^−^CD206^+^ cells observed between the M1+EVs+inhibitor and M1+EVs groups did not differ significantly (*P* > 0.05) (Fig. 3b). These observations suggest that *cel-let-7-5p* from *T. spiralis* NBL-EVs targets *C/EBPδ* to suppress the expression of the M1 macrophage marker CD86 on RAW264.7 cells.

### Nitric oxide content detection

**Fig. 4:**
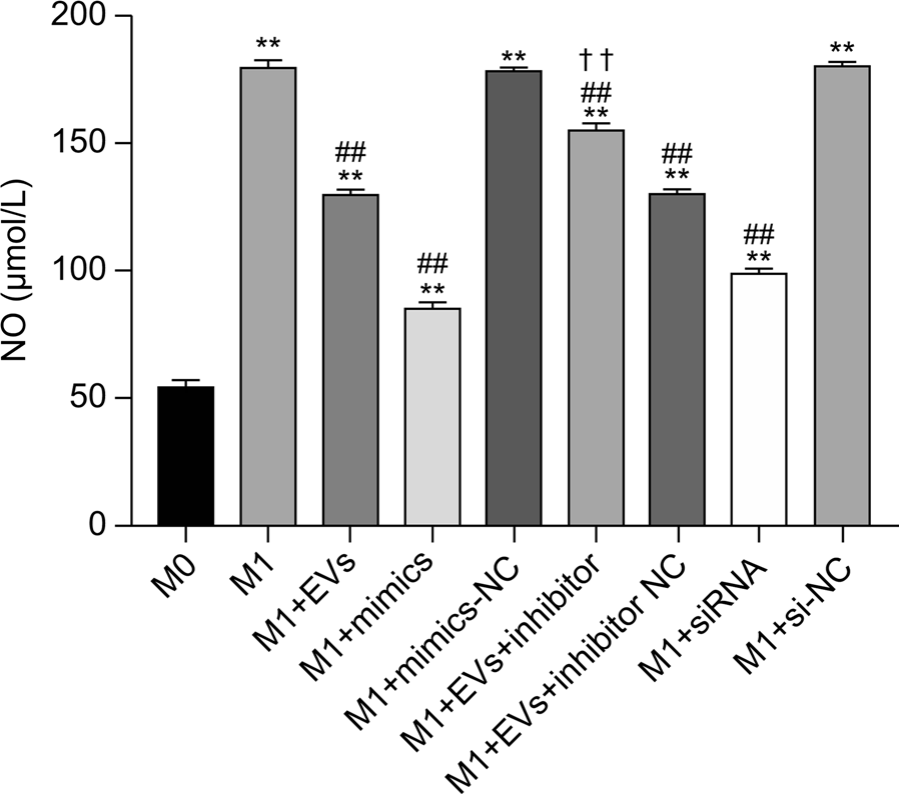
Statistical histogram of NO content in RAW264.7 groups. The data were analyzed from three independent experiments, and are expressed as the mean ± SD. **P < 0.01, compared with M0 group. ##*P* < 0.01, compared with the M1 group. ✝✝*P* < 0.01, compared with M1+EVs group. EVs, extracellular vesicles; NC, negative control; siRNA, small interfering RNA

NO assays showed that NO production in M1 cells was significantly increased when compared with that in M0 cells (*P* < 0.01). Treatment with EVs, mimics, EVs+inhibitor, EVs+inhibitor-NC, and siRNA resulted in a significant reduction in cellular NO levels when compared with the M1 group (*P* < 0.01). Moreover, NO levels in the M1+EVs+inhibitor group were significantly higher than those in the M1+EVs group (*P* < 0.01) (Fig. 4). These observations indicate that *cel-let-7-5p* from *T. spiralis* NBL-EVs could suppress NO release in the M1-like RAW264.7 cells by engaging *C/EBPδ*.

### ELISA to detect the release of cytokines

**Fig. 5:**
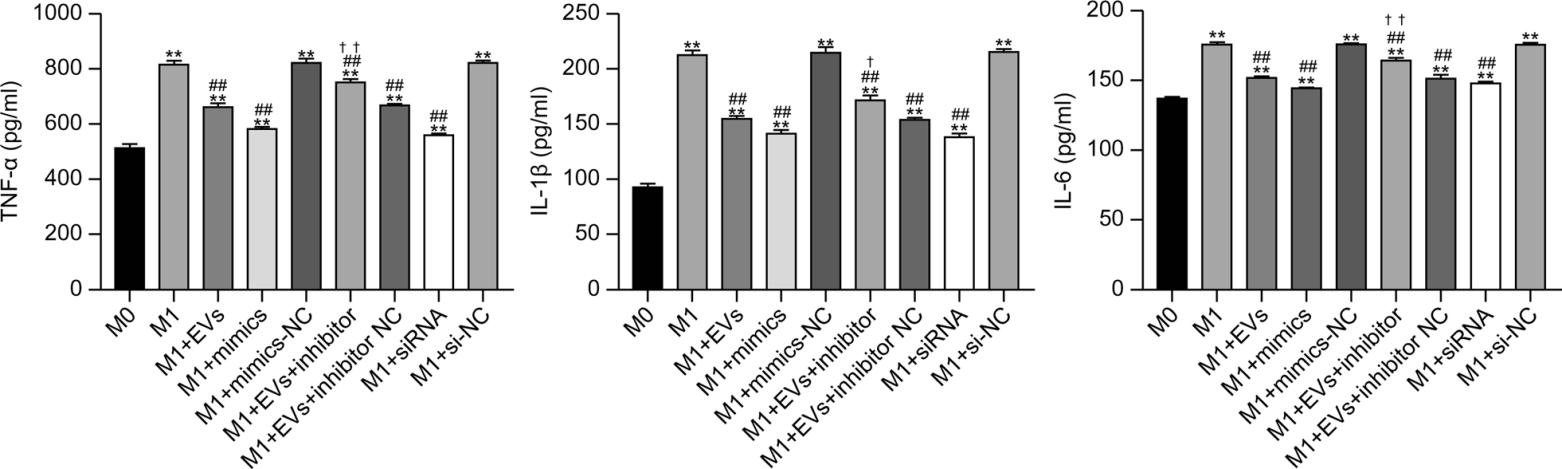
Statistical histogram of the cytokines release by RAW264.7 groups. The data were analyzed from three independent experiments, and are expressed as mean ± SD. **P < 0.01, compared with M0 group. ##*P* < 0.01, compared with the M1 group. ✝*P* < 0.05, ✝✝*P* < 0.01, compared with M1+EVs group. EVs, extracellular vesicles; NC, negative control; siRNA, small interfering RNA

The outcomes of the ELISA showed that RAW264.7 cells that were polarized to the M1 phenotype had significantly increased TNF-α, IL-1β, and IL-6 secretion when compared with the M0 group (*P* < 0.01). In contrast, the addition of EVs, mimics, and siRNA to RAW264.7 cells significantly reduced the secretion of TNF-α, IL-1β, and IL-6 when compared with the M1 group (*P* < 0.01). Moreover, the M1+EVs+inhibitor group displayed significantly increased secretion of TNF-α and IL-6, and a moderate increase in IL-1β, when compared with the M1+EVs group (*P* < 0.01 and *P* < 0.05, respectively) (Fig. 5). These data reveal that *cel-let-7-5p* from *T. spiralis* NBL-EVs can target *C/EBPδ* to inhibit the secretion of pro-inflammatory cytokines such as TNF-α, IL-1β, and IL-6 in M1 macrophages.

### RT-PCR analysis for the transcription of phenotypic-related molecules in macrophages

**Fig. 6:**
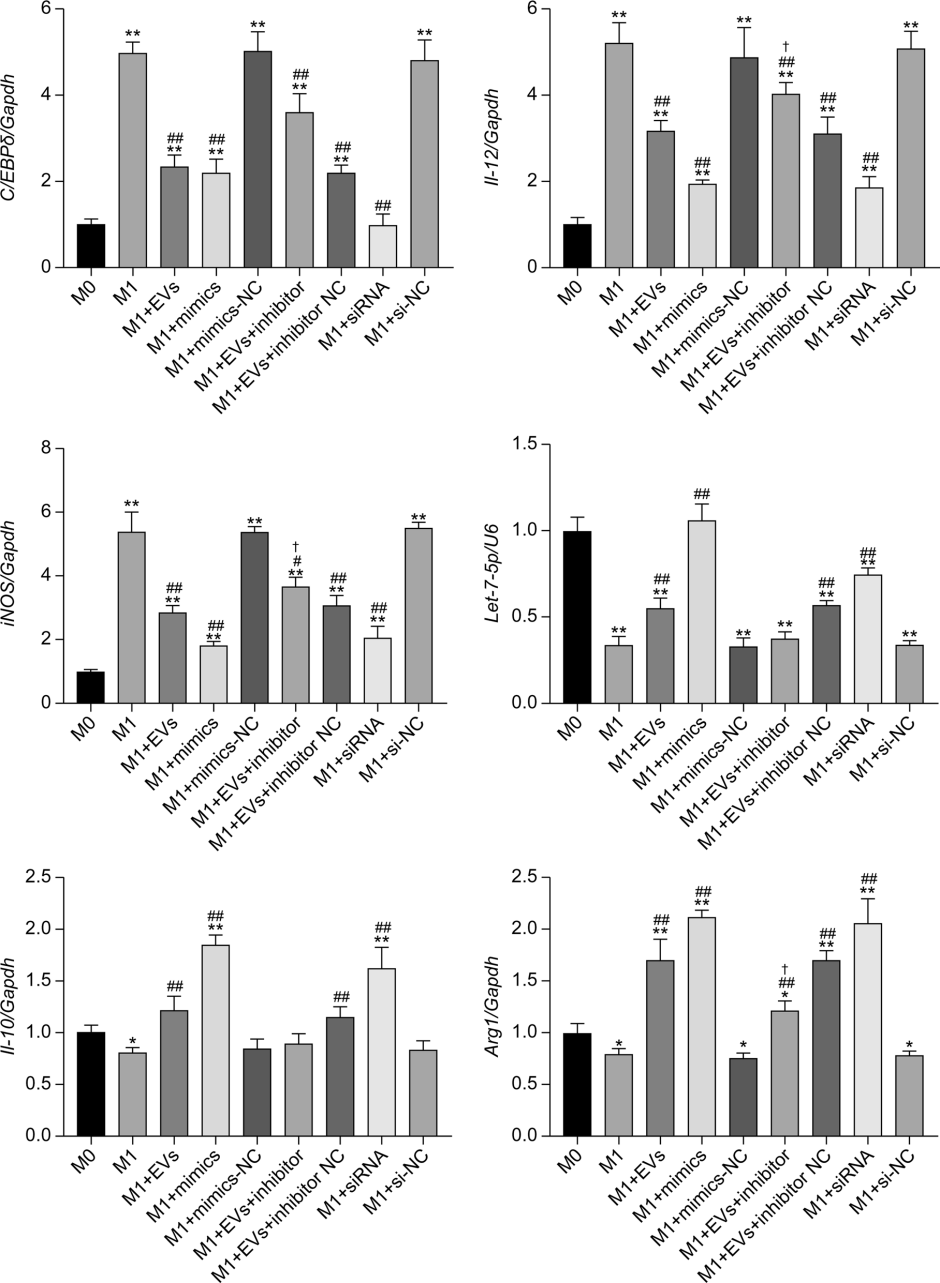
Statistical histograms of the transcription of characteristic molecules in RAW264.7 groups. The data were analyzed from three independent experiments, and are expressed as the mean ± SD. *P < 0.05, **P < 0.01, compared with M0 group. #*P* < 0.05, ##*P* < 0.01, compared with M1 group. ✝*P* < 0.05, compared with M1+EVs group. EVs, extracellular vesicles; NC, negative control; siRNA, small interfering RNA

RT-PCR data showed that M1 polarization significantly upregulated mRNA *C/EBPδ*, *IL-12*, and *iNOS* (*P* < 0.01) and significantly downregulated *let-7-5p* (*P* < 0.01) when compared with the M0 group. *IL-10* and *Arg1* levels also decreased significantly (*P* < 0.05). Compared with the M1 group, the addition of EVs, mimics, and siRNA significantly reduced *C/EBPδ*, *IL-12*, and *iNOS* transcription (*P* < 0.01) and increased *let-7-5p*, *IL-10*, and *Arg1*(*P* < 0.01). The M1+EVs+inhibitor group exhibited significantly higher *IL-12* and *iNOS* transcription than the M1+EVs group (*P* < 0.05); however, there was no significant change in* C/EBPδ* (*P* > 0.05), but *let-7-5p* decreased significantly (*P* < 0.05). Additionally, *IL-10* transcription remained unchanged (*P* > 0.05), whereas that of *Arg1* significantly decreased (*P* < 0.05) (Fig. 6). Overall, these results indicate that *cel-let-7-5p* from *T. spiralis* NBL-EVs targets *C/EBPδ* to inhibit the transcription of M1 macrophage markers, *IL-12*, and *iNOS*.

### Dual luciferase assay demonstrates that *C/EBPδ* is a target gene of the *T. spiralis* NBL-EVs cel-let-7-5p

**Fig. 7:**
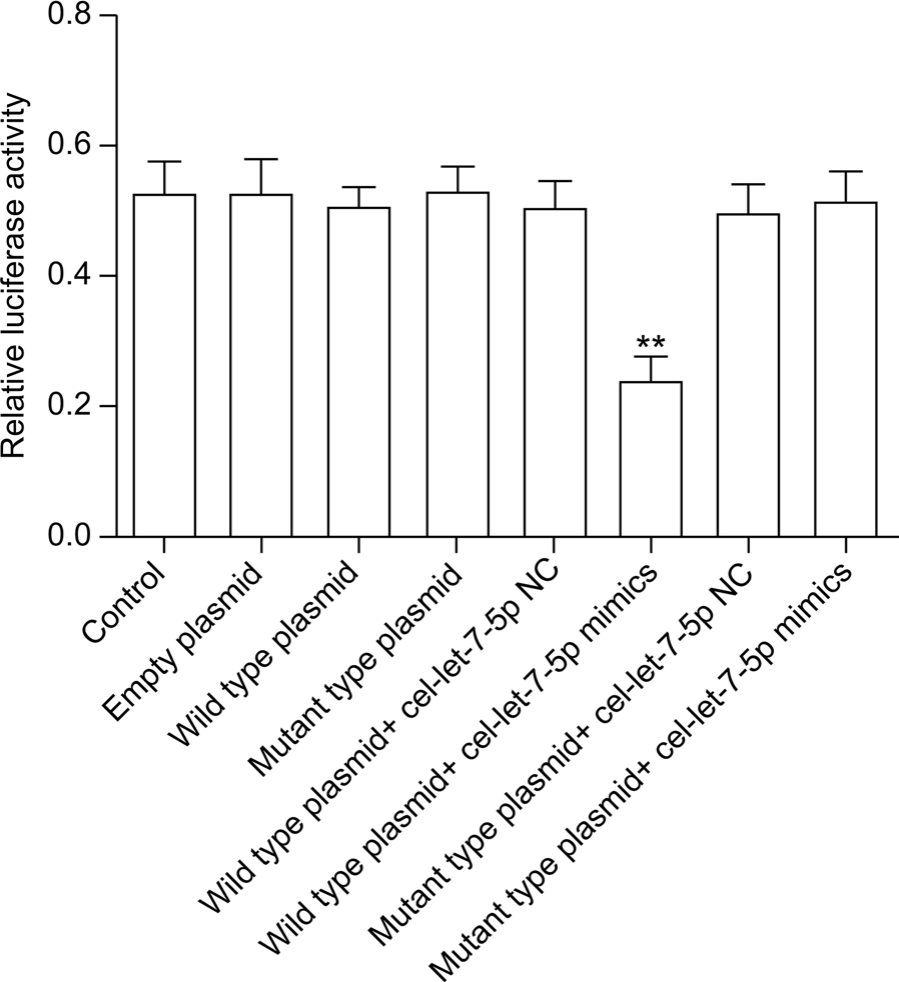
The results of dual luciferase reporter gene detection. The luciferase activity significantly decreased in the group co-transfected with the wild-type mRNA *C/EBPδ*-3′-UTR plasmid and *cel-let-7-5p* mimics compared with the control group (*P* < 0.01). The data were analyzed from three independent experiments, and are expressed as the mean ± SD. ** *P* < 0.01, compared with the control group

The dual luciferase reporter assay revealed that luciferase activity decreased considerably (*P* < 0.01) after co-transfection of 293 T cells with the wild-type mRNA *C/EBPδ-3*′-UTR vector and *cel-let-7-5p* mimics, when compared with the control. This outcome establishes that *C/EBPδ* is the target of *cel-let-7-5p* (Fig. 7).

### Western blot analysis for the detection of C/EBPδ protein expression

**Fig. 8:**
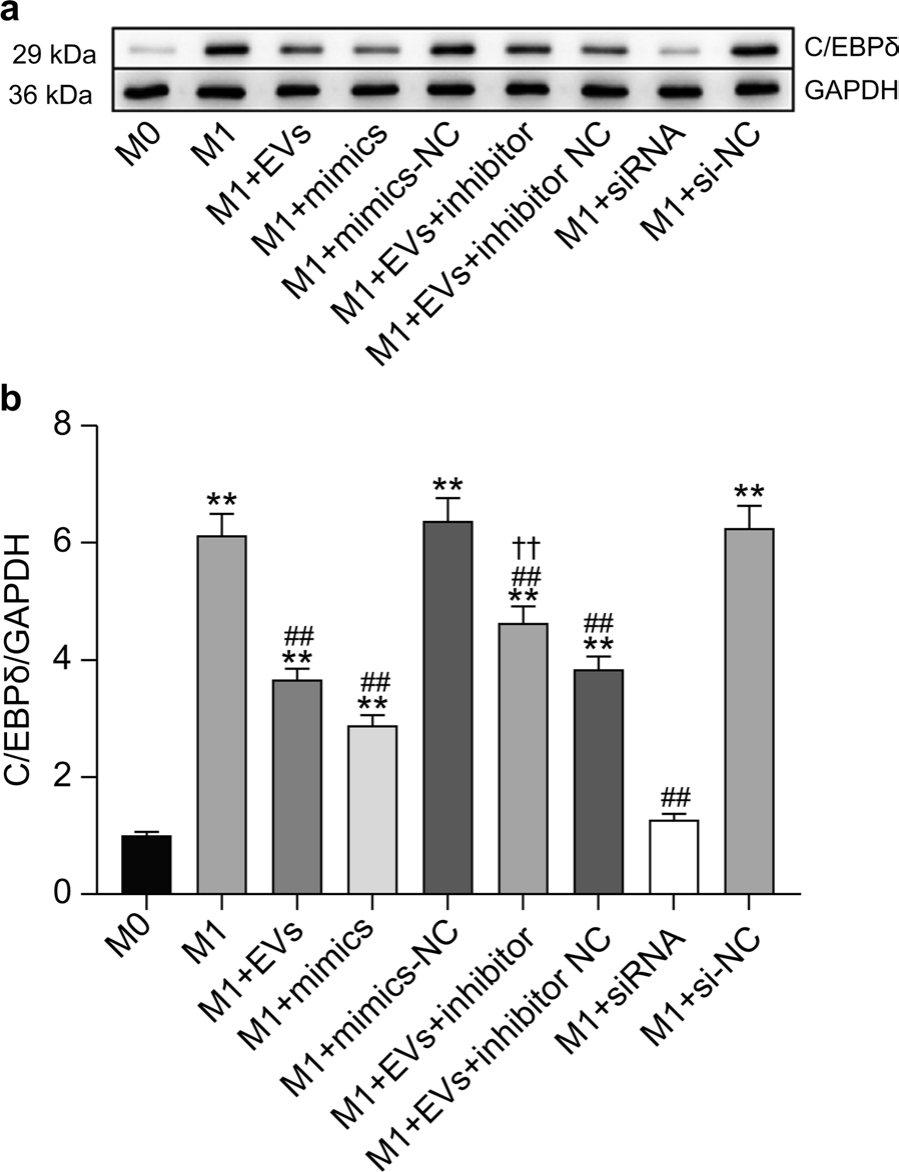
Western blotting results and statistical histogram of C/EBPδ expression in RAW264.7 groups. **a** Western blotting of C/EBPδ expression in RAW264.7 cells. GAPDH served as an internal control. **b** Intensity of C/EBPδ protein relative to that of GAPDH. The data were analyzed from three independent experiments, and are expressed as the mean ± SD. **P < 0.01, compared with M0 group. ##*P* < 0.01, compared with the M1 group. ✝✝*P* < 0.01, compared with M1+EVs group. EVs, extracellular vesicles; NC, negative control; siRNA, small interfering RNA

Western blot analysis showed that C/EBPδ protein levels were significantly increased in RAW264.7 cells after M1 polarization when compared with the M0 group (*P* < 0.01). In contrast, treatment with EVs, *cel-let-7-5p* mimics, and *C/EBPδ* siRNA caused a substantial decrease in C/EBPδ protein levels compared with the M1 group (*P* < 0.01). Furthermore, C/EBPδ protein expression in the M1+EVs+cel-let-7-5p inhibitor group was significantly higher than in the M1+EVs group in RAW264.7 cells (*P* < 0.01) (Fig. 8). These data show that *cel-let-7-5p* acts to suppress *C/EBPδ* expression.

## Discussion

EVs are vesicles with membrane structures that significantly contribute to various physiological and pathological processes by transporting their contents, such as DNA, lipids, metabolites, proteins, and small RNAs, into the target cells. The composition and function of miRNAs are currently a major focus of the studies on the composition of parasitic EVs [22]. Studies have shown that the genomic DNA, mRNA, and especially the non-coding RNA carried by EVs, including miRNAs, can provide a new gene exchange mechanism [23]. miRNAs, which are short (18–25 nucleotides) non-coding RNA molecules, modulate gene expression by binding to the 3′ UTR of their target mRNAs, thereby inhibiting translation or promoting mRNA decay. This process is guided by the highly conserved eight-base-pair seed region of miRNA in evolution [24].

*Let-7* was initially identified in *Caenorhabditis elegans*. It is essential for modulating the temporal aspects of stem cell proliferation and differentiation. The *let-7* family includes 12 mature miRNAs encoded by seven different genomic loci, and these sequences were identified across 101 species. The seed sequences of *let-7* and its family members are highly conserved across various organisms [25]. In parasitic organisms, there were early observations of *let-7* in EVs secreted by parasites such as *Dicrocoelium dendriticum*, *Brugia malayi*, *Trichuris muris*, and *Toxocara canis*, which influenced parasite development and modulated the host immune responses [26]. The role of *let-7* in regulating host macrophage responses mediated by parasitic EVs was recently highlighted. For example, *let-7-5p* from *T. solium* and its metacestode significantly reduces macrophage secretion of IL-16, IL-12, and TNF [20]. Additionally, *let-7-5p* present in EVs from *T. pisiformis* metacestode induces macrophage M2 phenotype polarization. It achieves this by inhibiting *C/EBPδ* expression [21] of *let-7-5p* from *Echinococcus multilocularis* EVs and modulates macrophage inflammatory response by downregulating key components of the IL-1α and LPS/toll-like receptor 4 pathways. It may also affect antigen presentation by upregulating CD40 expression [27]. Additionally, *let-7-5p* serves as a biomarker for the early diagnosis of *Echinococcus granulosus* infection in dogs [28]. In our previous study, we sequenced miRNAs from three instances of *T. spiralis* NBL-EVs and three corresponding instances of *T. spiralis* NBL. We identified 252 miRNAs, 40 of which showed significant differential expression (*P* < 0.05). Notably, compared with NBL, 10 miRNAs were significantly upregulated in NBL-EVs (*P* < 0.05), among which *cel-let-7-5p* was the most abundant, significantly exceeding the levels of the other nine miRNAs in NBL-EVs [17]. In *T. spiralis* ML-EVs, *cel-let-7-5p* is the third most abundant miRNA [19], possibly because of differing larval developmental stages. Evidence suggests that *let-7-5p*, derived from *T. spiralis* ML-EVs, facilitates the polarization of bone marrow macrophages toward the M2b phenotype and simultaneously inhibits fibroblast stimulation. This aligns with the anti-inflammatory response and suppression of Th1-type immune responses during the ML stage in the host, promoting wound healing and aiding *T. spiralis* in evading host immune clearance. Our findings revealed that *let-7-5p* in NBL-EVs inhibited M1 macrophages, which potentially contributed to the evasion of host defenses and dissemination of *T. spiralis* within the host during the early stages of infection.

In this study, we selected the *C/EBPδ* as the target gene for *T. spiralis* NBL-EVs *cel-let-5p* based on previous literature reports and verified it using dual luciferase experiments. *C/EBPδ*, or *nuclear factor IL-6β*, occupies the genetic position 8q11.21 and is classified within a superfamily of evolutionarily conserved transcription factors with a b-ZIP domain. These factors regulate various physiological functions such as inflammation control, cell cycle regulation, cellular differentiation, and metabolic activity [29]. The* C/EBP* family comprises six members, including *C/EBPα*, *C/EBPβ*, *C/EBPδ*, *C/EBP*ε, *C/EBPγ*, and *C/EBPζ*. They share substantial sequence similarity in their C-terminal regions, including a DNA-binding domain rich in basic amino acids, a leucine zipper dimerization domain, and a DNA-binding domain. Except for *C/EBPζ*, all family members bind to the same DNA recognition sites in the promoter regions of *C/EBP* target genes. Furthermore, transactivation domains located at the N-terminus mediate transcriptional regulation of *C/EBPγ*, with the capacity for both strong activation and dominant negative inhibition, influenced by interactions with repressor domains and basal transcriptional components [30]. Notably, *C/EBPβ* enhances the establishment and activity of M2 macrophages by regulating M2-specific gene expression, such as *Arg1*, *IL-10*, and macrophage scavenger receptor I (*MSR1*) [31]. Notably, the CREB-*C/EBPβ* signaling pathway regulates macrophage polarization by controlling the expression of M2-specific surface markers during *Mycobacterium tuberculosis* infection. Additionally, *C/EBPβ* activates the expression of M2-specific markers *Msrl*, *Arg1*, suppressor of cytokine signaling 3 (*Socs3*), interferon regulatory factor 4 (*Irf4*), and *IL-10*, among others, promoting macrophage polarization toward the M2 type [32]. Macrophages without *C/EBPδ* show significant defects in the production of inflammatory mediators, such as TNF-α, IL-6, and monocyte chemoattractant protein-1. *C/EBPδ* enhances macrophage inflammation by modulating multiple signaling pathways. Notably, research employing lentiviral transfection to knock down *C/EBPδ* in THP-1 cells revealed that after M1 induction, pro-inflammatory factors such as IL-1β, IL-6, iNOS, and TNF-α were decreased, in addition to reduced expression of the M1 surface marker CD80. This finding confirms the role of *C/EBPδ* in promoting M1 macrophage polarization [33]. Moreover, *C/EBPδ* interacts with NF-κB subunit p65 to enhance its transcriptional activity and elevate the expression of macrophage inflammation-related genes. It can also intensify the inflammatory response by activating MAPK and STAT3 signaling pathways [34]. The downregulation of *C/EBPδ* may also influence LPS-induced endoplasmic reticulum stress leading to increased calcium ions, which subsequently activates the p38 MAPK/CHOP/Fas pathway, inhibiting LPS-induced inflammation in mouse macrophages RAW264.7 [35]. In the present study, we synthesized *C/EBPδ* siRNA to knock down *C/EBPδ* in M1-polarized RAW264.7 cells. The results showed that M1-polarized RAW264.7 cells had reduced CD86 expression, intracellular NO synthesis, and transcriptional reduction of *IL-12* and *iNOS*, along with decreased *C/EBPδ* protein expression and reduced release of pro-inflammatory cytokines TNF-α, IL-1β, and IL-6. This indicates that knocking down *C/EBPδ* can inhibit the functionality of M1-polarized RAW264.7 cells.

During early *T. spiralis* infection (days 1–5), intestinal, mesenteric lymph node (MLN), and splenic macrophages exhibit a pro-inflammatory M1 phenotype critical for adult worm clearance. By day 15, splenic macrophages shift toward anti-inflammatory M2 polarization, with intestinal/MLN macrophages displaying mixed M1/M2 states; this transitions to dominant M2 activation in all tissues by day 30 [7]. Immune responses mediated by M2 macrophages are less effective in clearing adult worms but facilitate tissue repair and assist in ML escape. In co-culture studies of adult worms, ML, and NBL of *T. spiralis* with human THP-1 macrophages, the regulatory effects of excretory–secretory products (ES) from various *Trichinella* stages on macrophage activity were examined. The results showed that ES from ML possess anti-inflammatory potential and inhibit IL-1β, TNF-α, and IL-6 production by macrophages under LPS stimulation while reducing the expression levels of CCL3 produced by macrophages [36]. While M2 macrophage responses demonstrate insufficient efficacy in parasite clearance, they critically contribute to tissue regeneration and enhance nurse NBL and ML viability. Notably, the temporal coincidence between M1-to-M2 polarization in secondary lymphoid organs and the hematogenous dissemination phase of NBL suggests a potential mechanistic link. NBL-derived ES, particularly EVs systemically transported via hematogenous/lymphatic circulation, likely reach target organs during macrophage phenotypic transition. This polarization process, characterized by upregulated CD206/*Arg1*/*IL-10* expression concomitant with downregulation of CD86/*iNOS*/*IL-12*, attenuates pro-inflammatory responses through diminished IL-6/TNF-α/IL-1β secretion. Such immunomodulation establishes a permissive microenvironment that supports NBL migration and subsequent tissue colonization.

In vitro experiments demonstrated that NBL-EVs enriched with *cel-let-7-5p* significantly inhibit M1 macrophage functionality through targeted modulation of *C/EBPδ*. This inhibition manifested as reduced NO synthesis, downregulation of pro-inflammatory cytokine secretion (IL-6, TNF-α, and IL-1β), and promotion of phenotypic skewing toward M2 polarization. Furthermore, pharmacological inhibition of *cel-let-7-5p* within EVs substantially mitigated their suppressive effects on M1 RAW264.7 cells, particularly evidenced by diminished transcription of the M2 marker *Arg1*. Notably, neither CD206 expression nor *IL-10* transcription exhibited statistically significant alterations. This incomplete phenotypic conversion suggests that *cel-let-7-5p* functions as a partial regulator rather than the sole determinant of M2 polarization observed in vivo, implying potential synergistic interactions with other NBL-EV components—including auxiliary miRNAs, functional proteins, or bioactive lipids—that may collaboratively orchestrate immunomodulatory processes.

The partial restoration of M1 functionality following *cel-let-7-5 *pinhibition underscores the multifactorial regulatory mechanisms governing macrophage polarization by NBL-EVs. This finding necessitates comprehensive cargo profiling coupled with multi-omics integration to elucidate the combinatorial immunoregulatory networks. Current experimental constraints stem primarily from technical limitations in NBL-EVs isolation for in vivo validation. To address this knowledge gap, subsequent investigations should implement (1) fluorescent tracer-based quantification of NBL-EVs biodistribution in mesenteric lymph nodes and spleen, (2) comparative polarization analysis using EVs-depletion models and *cel-let-7-5p*-knockout infection systems, and (3) single-cell transcriptomic profiling to delineate macrophage functional heterogeneity during NBL dissemination. These proposed studies will critically evaluate the hypothesis that NBL-EVs mediate systemic immunosuppression to enhance parasitic survival, potentially revealing novel therapeutic targets for trichinellosis management.

## Conclusions

In conclusion, our findings indicate that *cel-let-7-5p* in *T. spiralis* NBL-EVs suppresses the activity of M1-polarized RAW264.7 macrophages by modulating *C/EBPδ*. This could help in understanding the shift in host macrophages from the M1 to the M2 phenotype during the larval migration phase of *T. spiralis* infection. This study enhances our understanding of the regulatory effects of *T. spiralis* NBL-EVs on host macrophages and provides a foundation for future research into the invasive mechanisms of *T. spiralis* larvae and the development of new therapeutic approaches, vaccines, and pharmaceuticals.

## Data Availability

No datasets were generated or analyzed during the current study.

## Abbreviations

ADCC: Antibody-dependent cytotoxicity
Arg1: Arginase-1
CCL: Chemokine (C–C motif) ligand
C/EBPδ: CCAAT/enhancer binding protein delta
COX-2: Cyclooxygenase-2
EVs: Extracellular vesicles
IFN-γ: Interferon-gamma
IL: Interleukin
IIL: Intestinal infective larval
LPS: Lipopolysaccharide
ML: Muscle larvae
miRNA: MicroRNA
NADPH: Nicotinamide adenine dinucleotide phosphate
NC: Negative control
NBL: Newborn larvae
NF-κB: Nuclear factor kappa B
NO: Nitric oxide
PPARγ: Peroxisome proliferator-activated receptor gamma
ROS: Reactive oxygen species
STAT6: Signal transducer and activator of transcription 6
TGF-β: Transforming growth factor beta
ML-EVs: Extracellular vesicles secreted by ML
NBL-EVs: Extracellular vesicles secreted by NBL
MISEV2023: Minimal information for studies of extracellular vesicles 2023
RAW264.7: Murine macrophage cell line
HEK293T: Human embryonic kidney cell line 293T
siRNA: Small interfering RNA
BSA: Bovine serum albumin
PBS: Phosphate-buffered saline
DMEM: Dulbecco’s modified Eagle’s medium
FBS: Fetal bovine serum
CCK-8: Cell counting kit-8
RT-PCR: Reverse transcription polymerase chain reaction
GAPDH: Glyceraldehyde-3-phosphate dehydrogenase
iNOS: Inducible nitric oxide synthase
Th1: T helper cell 1
TNF-α: Tumor necrosis factor alpha
UTR: Untranslated region
ES: Excretory–secretory products

## References

[1] World Health Organization. Risk-based examples and approach for control of *Trichinella* spp. and *Taenia saginata*. Rome: Food and Agriculture Organization; 2020.

[2] Falduto GH, Vila CC, Saracino MP, Calcagno MA, Venturiello SM. *Trichinella spiralis*: killing of newborn larvae by lung cells. Parasitol Res. 2015;114:679–85. 25416332 10.1007/s00436-014-4233-x

[3] Lazarov T, Juarez-Carreño S, Cox N, Geissmann F. Physiology and diseases of tissue-resident macrophages. Nature. 2023;618:698–707. 37344646 10.1038/s41586-023-06002-x PMC10649266

[4] Yunna C, Mengru H, Lei W, Weidong C. Macrophage M1/M2 polarization. Eur J Pharmacol. 2020;877:173090–8. 32234529 10.1016/j.ejphar.2020.173090

[5] Shapouri-Moghaddam A, Mohammadian S, Vazini H, Taghadosi M, Esmaeili SA, Mardani F, et al. Macrophage plasticity, polarization, and function in health and disease. J Cell Physiol. 2018;233:6425–40. 29319160 10.1002/jcp.26429 PMC13482560

[6] Lekki-Jóźwiak J, Bąska P. The roles of various immune cell populations in immune response against helminths. Int J Mol Sci. 2023;25:420–41. 38203591 10.3390/ijms25010420 PMC10778651

[7] Sun Q, Huang J, Gu Y, Liu S, Zhu X. Dynamic changes of macrophage activation in mice infected with *Trichinella spiralis*. Int Immunopharmacol. 2022;108:108716–26. 35344812 10.1016/j.intimp.2022.108716

[8] Zhang R, Zhang Y, Yan SW, Cheng YK, Zheng WW, Long SR, et al. Galactomannan inhibits *Trichinella spiralis* invasion of intestinal epithelium cells and enhances antibody-dependent cellular cytotoxicity related killing of larvae by driving macrophage polarization. Parasite (Paris Fr). 2024;31:6–21. 10.1051/parasite/2024002 PMC10854486 38334686

[9] Liu RD, Meng XY, Le Li C, Xu QY, Lin XZ, Dong BR, et al. *Trichinella spiralis* cathepsin L induces macrophage M1 polarization via the NF-κB pathway and enhances the ADCC killing of newborn larvae. Parasit Vectors. 2023;16:433–53. 37993938 10.1186/s13071-023-06051-1 PMC10666456

[10] Yan SW, Zhang R, Guo X, Wang BN, Long SR, Liu RD, et al. *Trichinella spiralis* dipeptidyl peptidase 1 suppressed macrophage cytotoxicity by promoting M2 polarization via the STAT6/PPARγ pathway. Vet Res. 2023;54:77–97. 37705099 10.1186/s13567-023-01209-2 PMC10500742

[11] Welsh JA, Goberdhan DCI, O’Driscoll L, Buzas EI, Blenkiron C, Bussolati B, et al. Minimal information for studies of extracellular vesicles (MISEV2023): from basic to advanced approaches. J Extracell Vesicles. 2024;13:e12404–87. 38326288 10.1002/jev2.12404 PMC10850029

[12] Sánchez-López CM, Trelis M, Bernal D, Marcilla A. Overview of the interaction of helminth extracellular vesicles with the host and their potential functions and biological applications. Mol Immunol. 2021;134:228–35. 33836351 10.1016/j.molimm.2021.03.020

[13] Diener C, Keller A, Meese E. Emerging concepts of miRNA therapeutics: from cells to clinic. Trends Genet. 2022;38:613–26. 35303998 10.1016/j.tig.2022.02.006

[14] Hill M, Tran N. miRNA interplay: mechanisms and consequences in cancer. Dis Model Mech. 2021;14:047662–70. 10.1242/dmm.047662 PMC8077553 33973623

[15] Chen L, Heikkinen L, Wang C, Yang Y, Sun H, Wong G. Trends in the development of miRNA bioinformatics tools. Brief Bioinform. 2019;20:1836–52. 29982332 10.1093/bib/bby054 PMC7414524

[16] Gao X, Yang Y, Liu X, Wang Y, Yang Y, Boireau P, et al. Extracellular vesicles derived from *Trichinella spiralis* prevent colitis by inhibiting M1 macrophage polarization. Acta Trop. 2021;213:105761–8. 33221281 10.1016/j.actatropica.2020.105761

[17] Liu Y, Cai YC, Chen JX, Chen S, Yu YF [Isolation, identification, and omics analysis of extracellular vesicles from Trichinella spiralis newborn larvae]. Chin J Parasitol Parasit Dis. 2024;42:225–33.

[18] Lee H, Han S, Kwon CS, Lee D. Biogenesis and regulation of the let-7 miRNAs and their functional implications. Protein Cell. 2016;7:100–13. 26399619 10.1007/s13238-015-0212-y PMC4742387

[19] Wu J, Liao Y, Li D, Zhu Z, Zhang L, Wu Z, et al. Extracellular vesicles derived from *Trichinella spiralis* larvae promote the polarization of macrophages to M2b type and inhibit the activation of fibroblasts. Front Immunol. 2022;13:974332–42. 36211336 10.3389/fimmu.2022.974332 PMC9532625

[20] Landa A, Navarro L, Ochoa-Sánchez A, Jiménez L. *Taenia solium* and *Taenia crassiceps*: miRNomes of the larvae and effects of miR-10-5p and let-7-5p on murine peritoneal macrophages. Biosci Rep. 2019;39:152–66. 10.1042/BSR20190152 PMC6863767 31694049

[21] Wang L, Liu T, Chen G, Li Y, Zhang S, Mao L, et al. Exosomal microRNA let-7-5p from *Taenia pisiformis* cysticercus prompted macrophage to M2 polarization through inhibiting the expression of C/EBP δ. Microorganisms. 2021;9:1403–14. 34209741 10.3390/microorganisms9071403 PMC8307393

[22] Sotillo J, Robinson MW, Kimber MJ, Cucher M, Ancarola ME, Nejsum P, et al. The protein and microRNA cargo of extracellular vesicles from parasitic helminths—current status and research priorities. Int J Parasitol. 2020;50:635–45. 32652128 10.1016/j.ijpara.2020.04.010

[23] Carrera-Bravo C, Koh EY, Tan KSW. The roles of parasite-derived extracellular vesicles in disease and host-parasite communication. Parasitol Int. 2021;83:102373–81. 33933651 10.1016/j.parint.2021.102373

[24] Kehl T, Backes C, Kern F, Fehlmann T, Ludwig N, Meese E, et al. About miRNAs, miRNA seeds, target genes and target pathways. Oncotarget. 2017;8:107167–75. 29291020 10.18632/oncotarget.22363 PMC5739805

[25] Roush S, Slack FJ. The let-7 family of microRNAs. Trends Cell Biol. 2008;18:505–16. 18774294 10.1016/j.tcb.2008.07.007

[26] Nawaz M, Malik MI, Hameed M, Zhou J. Research progress on the composition and function of parasite-derived exosomes. Acta Trop. 2019;196:30–6. 31071298 10.1016/j.actatropica.2019.05.004

[27] Jin X, Li Y, Yang X, Zheng Y. Modulatory effects of *Echinococcus multilocularis* emu-let-7–5p on the immunological functions of RAW264.7 macrophages. Front Vet Sci. 2021;8:663497–502. 33937384 10.3389/fvets.2021.663497 PMC8081858

[28] Celik F, Tektemur A, Simsek S. miRNA based biomarkers for the early diagnosis of *Echinococcus granulosus* in experimentally infected dogs. Vet Parasitol. 2023;324:110075–9. 38000178 10.1016/j.vetpar.2023.110075

[29] Sowamber R, Chehade R, Bitar M, Dodds LV, Milea A, Slomovitz B, et al. CCAAT/enhancer binding protein delta (C/EBPδ) demonstrates a dichotomous role in tumour initiation and promotion of epithelial carcinoma. EBioMedicine. 2019;44:261–74. 31078521 10.1016/j.ebiom.2019.05.002 PMC6603855

[30] Hartl L, Roelofs JJTH, Dijk F, Bijlsma MF, Duitman JW, Spek CA. C/EBP-family redundancy determines patient survival and lymph node involvement in PDAC. Int J Mol Sci. 2023;24:1537–51. 36675048 10.3390/ijms24021537 PMC9867044

[31] Lawrence T, Natoli G. Transcriptional regulation of macrophage polarization: enabling diversity with identity. Nat Rev Immunol. 2011;11:750–61. 22025054 10.1038/nri3088

[32] Sahu SK, Kumar M, Chakraborty S, Banerjee SK, Kumar R, Gupta P, et al. MicroRNA 26a (miR-26a)/KLF4 and CREB-C/EBPβ regulate innate immune signaling, the polarization of macrophages and the trafficking of *Mycobacterium tuberculosis* to lysosomes during infection. PLoS Pathog. 2017;13:e1006410. 28558034 10.1371/journal.ppat.1006410 PMC5466338

[33] Li M, Xiong YF, Huang XJ, Chen TA, Li JD. [CCAAT/enhancer binding protein δ inhibits invasion and metastasis of liver cancer by regulating M1 type macrophages polarization]. Zhonghua Gan Zang Bing Za Zhi. 2021;29:794–8. 34517463 10.3760/cma.j.cn501113-20200330-00149 PMC12813953

[34] Spek CA, Aberson HL, Butler JM, de Vos AF, Duitman JW. CEBPD potentiates the macrophage inflammatory response but CEBPD knock-out macrophages fail to identify CEBPD-dependent pro-inflammatory transcriptional programs. Cells. 2021;10:2233–47. 34571881 10.3390/cells10092233 PMC8470509

[35] Shahidullah A, Lee JY, Kim YJ, Halimi SMA, Rauf A, Kim HJ, et al. Anti-inflammatory effects of diospyrin on lipopolysaccharide-induced inflammation using RAW 264.7 mouse macrophages. Biomedicines. 2020;8:11. 31940845 10.3390/biomedicines8010011 PMC7168165

[36] Zawistowska-Deniziak A, Bień-Kalinowska J, Basałaj K. Regulation of human THP-1 macrophage polarization by *Trichinella spiralis*. Parasitol Res. 2021;120:569–78. 33415398 10.1007/s00436-020-07000-y

